# Quasi-Static Tensile Properties of Unalloyed Copper Produced by Electron Beam Powder Bed Fusion Additive Manufacturing

**DOI:** 10.3390/ma14112932

**Published:** 2021-05-29

**Authors:** Prithwish Tarafder, Christopher Rock, Timothy Horn

**Affiliations:** 1 Center for Additive Manufacturing and Logistics, Fitts-Woolard Hall, North Carolina State University, Raleigh, NC 27606, USA; ptarafd@ncsu.edu (P.T.); cdrock@ncsu.edu (C.R.); 2 Department of Mechanical and Aerospace Engineering, Engineering Building III, North Carolina State University, Raleigh, NC 27606, USA

**Keywords:** electron beam, powder bed fusion, copper, post-process, mechanical property, microstructure

## Abstract

Mechanical properties of powder bed fusion processed unalloyed copper are reported majorly in the as-fabricated condition, and the effect of post-processes, common to additive manufacturing, is not well documented. In this study, mechanical properties of unalloyed copper processed by electron beam powder bed fusion are characterized via room temperature quasi-static uniaxial tensile test and Vickers microhardness. Tensile samples were extracted both perpendicular and parallel to the build direction and assigned to three different conditions: as-fabricated, hot isostatic pressing (HIP), and vacuum annealing. In the as-fabricated condition, the highest UTS and lowest elongation were obtained in the samples oriented perpendicular to the build direction. These were observed to have clear trends between sample orientation caused primarily by the interdependencies between the epitaxial columnar grain morphology and dislocation movement during the tensile test. Texture was insignificant in the as-fabricated condition, and its effect on the mechanical properties was outweighed by the orientation anisotropy. The fractographs revealed a ductile mode of failure with varying dimple sizes where more shallow and finely spaced dimples were observed in the samples oriented perpendicular to the build direction. EDS maps reveal that grain boundary oxides coalesce and grow in HIP and vacuum-annealed specimens which are seen inside the ductile dimples and contribute to their increased ductility. Overall, for the post-process parameters chosen in this study, HIP was observed to slightly increase the sample’s density while vacuum annealing reduced the oxygen content in the specimens.

## 1. Introduction

The benefits of complex geometries, reduced assemblies, and rapid build time attributed to powder bed fusion (PBF) additive manufacturing (AM) have recently been extended to unalloyed copper for a variety of applications requiring very high thermal and electrical conductivity, complicated geometries, and extensive processing routes. Examples include, but are not limited to, electric motors [1], vacuum electronics [2,3,4,5,6], and heat exchangers [7,8]. However, compared to other common AM metals, the feasibility of PBF-AM of copper has only recently been demonstrated, and for all intents and purposes is at a relatively early stage of developmental maturity. Within the current literature, studies have typically focused on process parameter development with the predominant metrics investigated being material density and, to a lesser degree, material purity. The documented AM processing space is primarily driven by relatively small, prismatic geometries. As a result, the very basic property characterizations are found only sporadically in the scientific knowledge base, and then only a limited number of these studies have reported mechanical property data for AM fabricated copper; the highlights of which are summarized in Table 1 [9,10,11,12,13,14].

In part, the lack of component-level data can be attributed to the significant processing challenges associated with PBF-AM of copper. Recent data from the literature suggest that the feasible AM process window for both laser PBF (L-PBF) and electron beam PBF (EB-PBF) is effectively narrow and highly sensitive to thermal boundary conditions and geometric influences within a layer. These effects are exacerbated by the high thermal conductivity of the material [5,14]. Therefore, while AM-produced copper components exhibiting complex geometries have been reported, the process parameter and properties data are typically associated with small prismatic specimens.

For L-PBF, the high thermal conductivity, high reflectance (for typical ~1µm wavelengths used in AM), and oxidation are well documented and often cited as the most significant process barriers for fabrication [8,15]. Recent work with high-power lasers (400–1000 W) [10,14,16,17,18], shorter laser wavelengths in the visible spectrum [19,20,21,22], and micro-alloying [23,24] have incrementally improved processing, material density, and have shown promising properties. EB-PBF of unalloyed copper generally circumvents many of the barriers faced by L-PBF techniques. The energy coupling between the beam and the powder is significantly higher [5], the mid-level vacuum environment (~2 × 10^−3^–2 × 10^−6^ mBar) contributes toward minimizing oxygen pickup, and the high beam power and scan speeds facilitate efficient bed preheating to reduce global thermal gradients [8,25], although the latter results in powder sintering which can complicate powder removal.

As expected, the reported ultimate tensile strength (UTS) and yield data are skewed by sample density. This is notably evident in the L-PBF studies, where the reported density has a wide range. Lykov et al. [9] reported a UTS of 149 MPa with a relative density of 88.1%, whereas Yan et al. [10] and Jadhav et al. [14] fabricated copper with a relative density up to 99.1% and 99.3% that resulted in an UTS of 248 and 211 MPa, respectively. For EB-PBF, however, most of the reported relative densities exceeded 99.5% [11,12,26,27,28]. Guschlbauer et al. reported a maximum UTS of 231.6 MPa tested transverse to the build direction; the lowest UTS reported in the same study was 181.3 MPa oriented parallel to the build direction [11]. It should be noted that these data, for both EB-PBF and L-PBF, are typically extracted from sub-size tensile specimens where the influence of grain size, compositional contamination, or a particularly large defect will have a significant effect.

Apart from powder bed fusion AM processes, Kumar et al. have reported UTS values of 176.4 ± 6.5 MPa with 67.2 ± 2.2% elongation for copper produced using a binder jet (BJ) AM that was sintered in a reducing atmosphere and subsequently subjected to hot isostatic pressing (HIP) at 1075 °C [13]. While of lower purity and subject to contamination introduced by the binder material, the microstructure reported by the authors is refined and equiaxed as a result of the solid-state processing. This is in contrast to the directional microstructure typical of the powder bed fusion process of unalloyed copper reported in the literature.

Alongside the solid density, the effect of oxygen content on ductility, particularly outside the range of what would typically be considered pure copper, is well documented [5]. Myers and Blythe reported that in cast copper, ductility was maintained up to an oxygen content of 280 wt. ppm, beyond which it led to a significant loss of ductility [29], in accord with the findings of Guschlbauer et al. [11] that attributed copper oxides acting as stress concentration sites during the tensile test of EB-PBF processed copper. Further, with the exception of the data presented by Ledford et al. [1,26], which do not include any mechanical properties, the EB-PBF automatic control parameters were explicitly disabled. While open-loop on most EB-PBF systems, such parameters serve to regulate beam speed and power to maintain stable melt pool size and powder bed temperature as part geometries evolve. It is therefore unclear how the reported properties data translate to more complicated geometries and beam control strategies. Therefore, exploration of mechanical properties of AM copper subjected to post-processing steps common to AM such as HIP and annealing could have the potential to widen the effective processing space and enable broader geometric variation with AM copper components. In the present study, results of room temperature quasi-static uniaxial tensile test and microhardness measurements were recorded for copper specimens produced via EB-PBF with automatic control settings enabled across multiple runs with X-oriented (perpendicular to build direction) and Z-oriented (parallel to build direction) specimens. A subset of the samples was subjected to HIP to explore the influence of densification on residual micro porosity while another group of samples from the same builds were subjected to vacuum annealing which is known to reduce oxygen content through diffusion and evaporation [30]. The remaining specimens were tested in the as-fabricated condition.

## 2. Materials and Methods

### 2.1. Powder Feedstock

Nitrogen gas-atomized copper powder screened to −270/+635 mesh was obtained from Sandvik Osprey (Sandvik Osprey, Neath, UK). The initial oxygen content of the powder was 457 wt. ppm, measured using inert gas fusion (LECO OH836, copper protocol, LECO, St. Jospeh, MI, USA). Powder particle size measurement was carried out on a Microtrac S3500 (Microtrac Inc., Montgomeryville, PA, USA) using a laser diffraction technique. The unalloyed copper powder chemistry was obtained using the inductively coupled plasma mass spectroscopy (ICP-MS, Laboratory Testing Inc., Hartfield, PA, USA) method. Powder morphology was observed using a JEOL 6010LA scanning electron microscope (JEOL, Peabody, MA, USA).

### 2.2. Sample Fabrication

**Figure 1:**
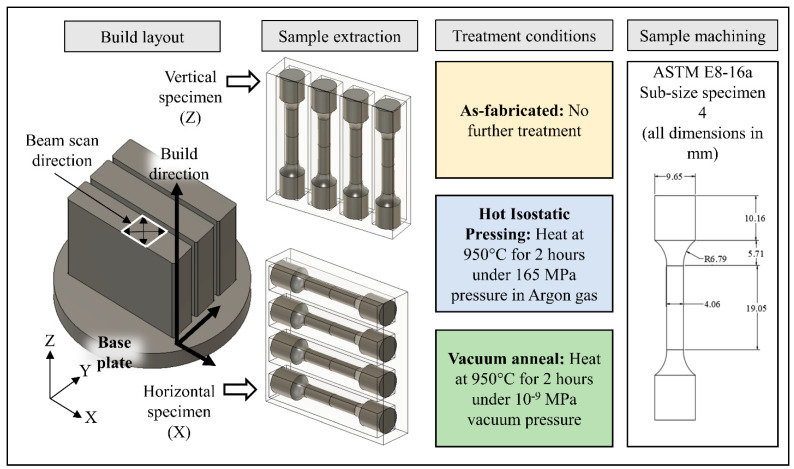
Overall experimental framework highlighting the build layout and sample orientations, treatment conditions, and the ASTM Standard design of the test coupons used in the study.

Five builds, each containing three rectangular blocks measuring 58.5 mm × 12.7 mm × 58.5 mm, were fabricated upon an 86 mm diameter and 40 mm thick oxygen-free copper base plate using a customized Arcam A2 EB-PBF system (Software version 3.2, SP2, Arcam AB), the details of which are described by Ledford et al. [31]. The nominal layer thickness was 40 µm. The powder bed temperature was measured by a thermocouple affixed to the bottom of the build substrate and was observed to be stable throughout the build duration within a range of 290–320 °C. The vacuum level inside the chamber was maintained at 5 × 10^−4^ mbar. Table 2 and Table 3 show the process parameters used to fabricate the samples. As illustrated in Figure 1, the rectangular blocks were oriented 45° about the z-axis. The beam raster patterns were oriented parallel and perpendicular to the x-axis and rotated 90° each layer. The blocks were melted “model-wise,” one by one so that each experienced nearly uniform scan line length (shown schematically on the front block within the build layout) and thermal history. Upon completion, the build volume was allowed to cool to below 30 °C prior to removal from the build chamber. The powder cake surrounding the parts was removed manually in an argon-filled glove box maintained with an atmospheric oxygen content below 10 wt. ppm. The powder was passed through a 70-mesh screen to remove large agglomerations and blended with the primary batch prior to each reuse. Powder size and oxygen content of both the reused powder and fabricated solids were measured after each build.

Figure 1 illustrates the experimental design. The orientation of the tensile bars within each block was randomly assigned subject to the constraint that only specimens with a single orientation could be extracted from any one block. As illustrated in Figure 1, each as-fabricated block was cut into four tensile blanks using a bandsaw assigned as *Z* when the blank was cut with the long sample axis parallel to the build direction and *X* when the long sample axis was perpendicular to the build direction using the ASTM nomenclature [32]. Each of the tensile blanks was then randomly assigned to one of three treatment groups. Samples in the as-fabricated group were tested without treatment, samples from the HIP group were subjected to HIP using a QIH15LMURC high pressure furnace (Quintus Technologies, Lewis Center, OH, USA) with a heating rate of 10 °C/min to 950 °C, a dwell of 2 h, and cooling at a rate of 10 °C/min. The pressure is proportional to the temperature, and at 950 °C was 165 MPa. Several preliminary experiments were conducted at Quintus Technologies to identify this particular parameter that yielded high densities. Samples assigned to the vacuum-annealing group were subjected to the same temperature profile in a tube furnace (Thermo-Scientific Lindberg/Blue M HTF55322C, Hillsboro, OR, USA), but under vacuum at 5 × 10^−5^ mbar (5 × 10^−9^ MPa). After treatment, all samples were machined into ASTM subsize specimens (specimen type 4) [33]. The surface finish of the machined specimens in the gauge region measured an average of 16 µin *R_a_* using a stylus profilometer (Mitutoyo Surftest SJ210, Aurora, IL, USA).

### 2.3. Quasi-Static Uniaxial Tensile Test Procedure

**Figure 2:**
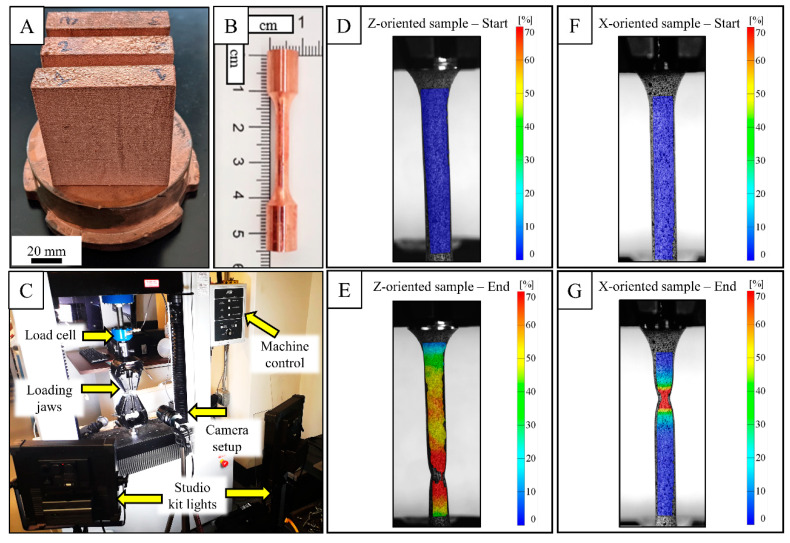
Fabricated blocks on a copper substrate (**A**), Machined tensile sample according to [33] (**B**), Overall tensile test setup showing the arrangement of DIC camera and lighting kits (**C**), Strain mapping of a Z- and X-oriented vacuum-annealed tensile sample in GOM Correlate software in the beginning (**D**,**F**) and the end (**E**,**G**) of the test, respectively.

Figure 2A–G show a photograph of the tensile testing setup. Tensile specimens harvested from the fabricated blocks (Figure 2A) were tested to failure after respective treatment conditions at room temperature using an ATS 1620C universal testing machine (Applied Test Systems, Inc., Butler, PA, USA) with wedge grips and a constant crosshead speed of 0.254 mm/min using a 5 kN load cell. Strains were calculated using digital image correlation (DIC). One face of the gauge section of each sample was painted with white background with black speckles. As the specified ductility of the dried paint material is lower than that of pure annealed copper, tests were conducted while the speckle paint was still wet. Images were acquired at 30 frames per minute using a 12.3 MP FLIR Grasshopper camera (FLIR, Wilsonville, OR, USA) under appropriate illumination conditions obtained using two F&V K4000S bi-color studio lighting kits (F&V, Mundelein, IL, USA), illustrated in Figure 2C. Strain field mapping and elongation measurement were carried out using GOM Correlate software (2018 Hotfix 6, Rev. 117418), where selected speckles were tracked for making strain measurements. Figure 2D–G show an example of such strain field mapping of a Z- and X-oriented vacuum-annealed specimen at the beginning and end of the test. The yield stress was calculated from a 0.2% offset from the linear portion of the stress-strain curve. The mechanical properties results were compared using ANOVA (JMP Pro 15. Ink) with pooled variance. Prior to analysis, the data were tested for normality using the Shapiro-Wilk test with the α-value of 0.05.

### 2.4. Metallographic Analyses and Microhardness Measurements

The grip section of each tensile bar was cut perpendicular to the longitudinal axis approximately 10 mm from each end. These samples, in turn, were sectioned longitudinally with a low-speed diamond saw (Buehler Isomet, Lake Bluff, IL, USA). The cut face was potted in phenolic resin and progressively ground using 400, 600, 1000, 2000 and 4000 grit SiC paper with water lubricant. Samples were polished with 1-micron, 0.3-micron, and 0.05-micron alumina slurry, and etched with Ammonium Persulphate solution by submersion/swabbing for 10 s and rinsing to reveal the grain structure. Samples were indented for Vickers microhardness measurements in a LECO M400 hardness tester (LECO, St. Joseph, MI, USA) under 100 gf load for a dwell time of 10 s. A total of ten indentations, 500 µm apart, were made along the direction of tension for both types of sample orientation, and the average values are reported. The sectioned grip end that was not used for metallographic analysis was used to measure the density of the sample using gas pycnometry (Quantachrome Ultrapyc 1200E according to [34]). Approximately 1 +/− 0.0002 g of the same was sectioned longitudinally for oxygen content measurement with inert gas fusion (as mentioned in Section 2.1). After tensile testing, a thin slice of the grip end along the build direction was cut and polished for as-fabricated, HIP, and vacuum-annealed X-oriented samples for electron backscatter diffraction (EBSD) which was performed on the FEI Quanta 3D Field Emission Gun (Thermo Fisher Scientific, Hillsboro, OR, USA) equipped with an Oxford Instruments EBSD detector on a 3 mm × 3 mm area on samples extracted with a X-sample orientation. Inverse pole figure (IPF) maps and pole figures were generated with Channel 5 Tango and Mambo software, respectively. The fracture surfaces of the tensile specimens were observed with the JEOL 6010LA SEM. Characterization of grain boundary oxides was carried out with STEM-EDS using a ThermoFisher Talos F200X TEM (Thermo Fisher Scientific, Hillsboro, OR, USA). The TEM samples were prepared with a Hitachi Ar-blade 5000 ion mill for 15 min prior to sample lift out, thinning, and mounting with the FEI Quanta 3D FEG.

## 3. Results and Discussion

### 3.1. Powder Characterization

**Figure 3:**
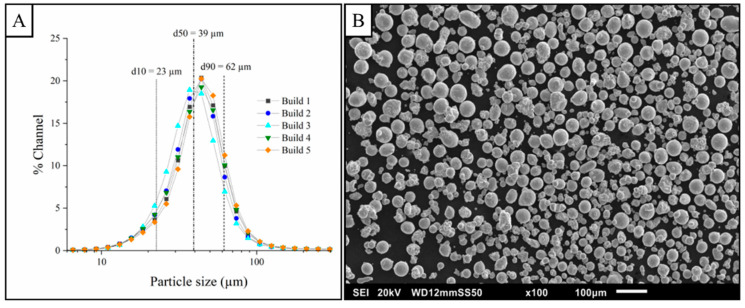
Particle size distribution of feedstock unalloyed copper powder used across the builds (**A**), and SEM image of the powder prior to the first build (**B**); scale bar shows 100 µm.

Figure 3A depicts the particle size distribution of the powder used in this study with a d10–d90 size range of 22–63 µm and d50 value of 39 µm and demonstrates no significant change in particle size with reuse over the course of this study. Table 4 shows the chemistry of the initial feedstock as measured by ICP-MS where no major contamination was detected. The oxygen content of the powder does show an increase during the study from an initial 457 ppm to a final value of 630 ppm after the fifth run. While the raw material used to produce the powder was oxygen free (class 1 oxygen free electrolytic grade (OFE) copper [35], C10100) with <5 ppm oxygen, the measured level of oxygen in the powder is similar to that of electrolytic tough pitch (ETP) C11000 with approximately 400–600 ppm oxygen [5], and can be attributed to the thin layer of non-passivating cuprous oxide and the high specific surface area of AM powders. This oxygen pickup can be difficult to avoid in practice and originates in the screening of powder to size fractions suitable for AM as well as from build-to-build powder handling. It is likely that special handling and monitoring or techniques to reduce surface oxides may be required. While the oxygen is elevated in the feedstock powder, it is within the range of recent reports for 3D-printed copper powder [14,31,36], where no AM-related study has reported oxygen values for copper powder that approach the values of C10100.

The SEM image shown in Figure 3B illustrates chiefly spherical morphology with scattered satellite particles attached to the powder surfaces, a typical feature of gas atomized metal powder. While the powder distribution is lower than that typically used by EB-PBF (45–106 µm), no detrimental effects on powder bed formation or electrostatic charging due to the size distribution were observed. It should also be noted that the higher electrical conductivity of copper mitigates the charge-induced scattering common in other AM materials [37]. Horn et al. [6] have also previously demonstrated improvements in the surface finish of EB-PBF-produced copper associated with finer powder size distributions.

### 3.2. Processing Space of Copper in EB-PBF

**Figure 4:**
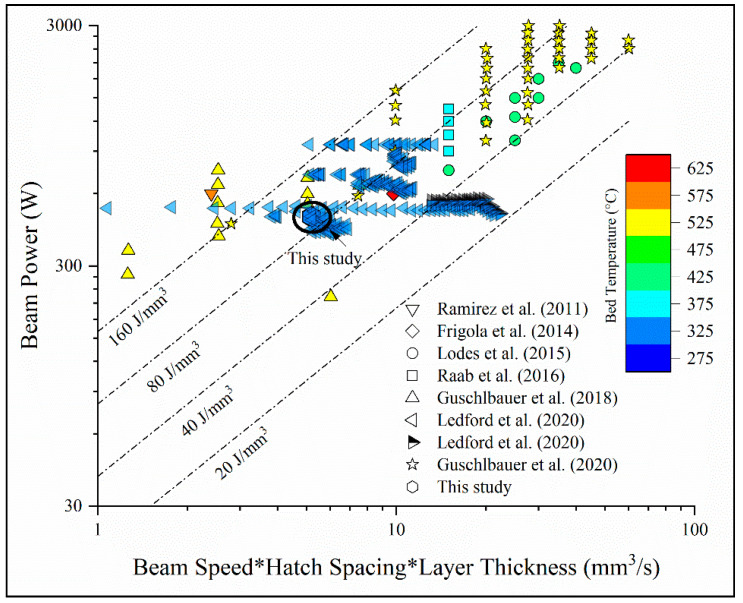
Processing space of EB-PBF fabrication of unalloyed copper by taking the process parameters used in the literature. Major observations of such studies are highlighted along with reported solid densities in Table 1.

Figure 4 illustrates the processing space of unalloyed copper via the EB-PBF method reported in the literature and used in this study. A closer look at Figure 4 and properties reported in Table 1 suggests that the reported EB-PBF processing space is indeed narrow and is sensitive to powder characteristics like size distribution and oxygen content that collectively lead to significant variation in properties. As seen in Figure 4, Ramirez et al. [36] studied EB-PBF of an oxidized copper powder with 99.8% purity that was processed at a high substrate temperature and a low beam speed, ultimately leading to an increased hardness of 88 HV in the solids. Lodes et al. and Raab et al. reported a stable process window at substrate temperatures close to 400 °C for fabricating >99.9% pure copper articles at various scan speeds and melt currents resulting in average relative densities greater than 99.95% [27,28]. Guschlbauer et al. [11,12] expanded this processing space and reported mechanical properties data for two different powder types (pure and oxidized), three build orientations (vertical, horizontal, and 45°), and variations in process parameters. The work by Guschlbauer et al. also highlights, in a more systematic way than previous studies, the influence of copper oxides on the strength and ductility in as-fabricated conditions. However, the processing space reported by Ledford et al. [1,26] is in contrast with others as the substrate temperature is relatively low because of the low initial oxygen content of the powder that tends to sinter more easily at high temperature preheat settings. Moreover, in literature it is generally seen that oxygen content in the powder feedstock ranges from 40 wt. ppm [31] to ~2000 wt. ppm [36], and warrants for different processing conditions to be adopted. Small powder size distribution and a powder purity of ~99.95% used in this study led to the use of a low substrate temperature, and as a result, low preheat and melt currents to avoid any flowability issues during the raking process. In terms of volumetric energy density (*E*), defined as:

(1) $$E=\frac{P}{vht}$$

where *P* is the beam power in J/s, *v* is the beam velocity in mm/s, *h* is the scan line offset in mm and *t* is the layer thickness in mm, the used parameter set resulted in an average volumetric energy density of 92.3 J/mm^3^, as shown in Figure 4.

### 3.3. Material Characterization 

**Figure 5:**
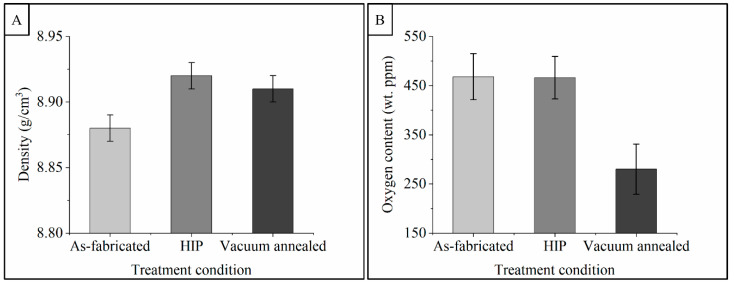
Plots illustrating the effect of treatment condition on the density (**A**) and oxygen content (**B**) of copper produced by EB-PBF. Error bars indicate a 95% confidence interval based on the pooled variance of all samples.

Figure 5A reports the density of samples from each treatment condition. The error bars shown represent a 95% confidence interval based on pooled variance of all samples. Within each treatment group no difference in specimen density was detected based on sample orientation, build number, or location within the build. Mean density of the specimens ranges from 8.88 to 8.92 g/cm^3^ (99.4% to 99.9% of theoretical). Specimens subjected to both vacuum-anneal heat treatment and HIP exhibited a slightly higher density (8.91 ± 0.01 and 8.92 ± 0.01 g/cm^3^, respectively) compared to as-fabricated samples (<*p* = 0.0001), however no significant difference in density was detected between the HIP and vacuum-annealed specimens (*p* = 0.084). It should also be noted that the overall densification was relatively small from the as-fabricated to treated conditions (only 0.45%), as the as-fabricated density was already 8.88 ± 0.1 g/cm^3^.

Figure 5B shows the effect of oxygen on post-treatment. As noted previously, the oxygen content of the powder trended upward from build 1 to build 5; within each build there was no difference between the oxygen content of the powder and the solid specimens prior to treatment, and no difference in oxygen content was detected as a function of sample orientation. The as-fabricated specimens and specimens subjected to HIP showed no difference in oxygen content across all builds with average values of 468 ± 47 wt. ppm and 466 ± 43 wt. ppm, respectively. Specimens subjected to the vacuum-annealing treatment were significantly different than the other groups, with an average oxygen content of 280 ± 51 wt. ppm. With a vacuum-annealing heat treatment of 950 °C for 12 h, EB-PBF-produced copper specimens were previously shown to be reduced from 500 wt. ppm to 30 wt. ppm [5]. Nieh and Nix had previously shown that annealing copper at 950 °C under a vacuum environment resulted in the dissociation of copper oxide and subsequent evaporation from the surface [30]. In this study, the vacuum-annealing time was limited to match the time and temperature profile of the HIP treatment. These data support previous results suggesting that vacuum annealing may be an effective method of removing oxygen from AM copper. Based on the low diffusion coefficient of oxygen in copper, however, wall thickness may be a practical limitation where high isothermal holding time and increased processing temperature will be required to promote the oxide dissociation [38].

**Figure 6:**
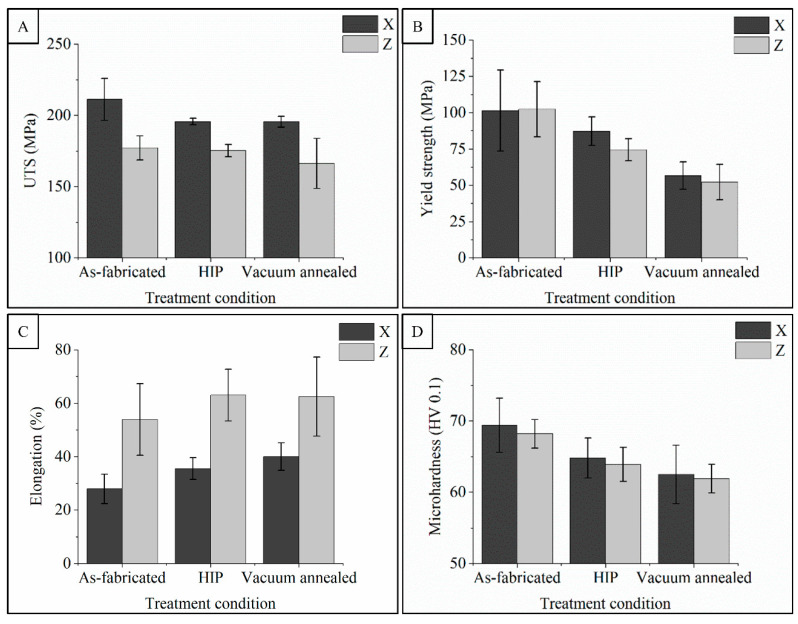
Mechanical properties summary and selected statistical observations; ultimate tensile strength (**A**), yield strength (**B**), elongation to failure (**C**), Vickers microhardness (**D**). Error bars indicate a 95% confidence interval based on the pooled variance of all samples.

The plots in Figure 6 report the measured mechanical property results from the experiments, including UTS, yield, elongation, and microhardness in the as-fabricated condition and after post-processing conditions for each orientation. ANOVA failed to detect any significant difference in UTS, yield, or elongation within each treatment group associated with the sample batch number (*p* > 0.15), the block number from which the samples were harvested (*p* > 0.41). These trends are observed in Figure 6 where the mechanical properties data are plotted with test orientation and treatment condition.

The X-oriented specimens exhibited higher UTS compared to Z-oriented specimens for all three processing conditions: as-fabricated with 211.2 ± 14.7 and 177.2 ± 8.5 MPa (*p* = 0.0003), HIP with 195.7 ± 2.2 and 175.3 ± 4.3 MPa (*p* < 0.0001), and vacuum-annealed with 195.5 ± 3.8 and 166.2 ± 17.6 MPa (*p* = 0.0007), respectively (see Figure 6A). The average yield stress, measured from a 0.2% offset from the linear portion of the stress-strain curve, was observed to vary between as-fabricated, HIP, and vacuum-annealed specimens regardless of orientation, as illustrated in Figure 6B. No significant difference was detected in the measured yield stress between the X and Z specimens in the as-fabricated condition with 101.4 ± 27.9 and 102.5 ± 19 MPa (*p* = 0.931), respectively, after HIP 87.3 ± 9.9 and 74.5 ± 7.6 MPa (*p* = 0.0475), or after vacuum-annealing, 56.7 ± 9.4 and 52.2 ± 12.3 MPa (*p* = 0.42). The as-fabricated samples consistently had the highest yield values, averaging just over 100 MPa, but also the widest variance due most likely to residual defects such as localized unhealed layer defects. The HIP treatment had an average midrange value for yield stress, ~20% lower than the as-fabricated condition, and a reduced scatter for both orientations (*p* = 0.0016). Consistently low yield stress values were observed in the vacuum-annealed samples, ~46% lower than the as-fabricated condition (*p* < 0.0001) and ~32% lower than the HIP condition (*p* < 0.0001).

While the strength values were observed to be influenced by harvest orientation (UTS) and post-treatment condition (0.2% yield), the most obvious property affected was elongation, as shown in Figure 6C. On average, the elongation of the Z-oriented specimens (53.9 ± 13.4%) was 93% higher than the X-oriented specimens (27.9 ± 5.5%) in the as-fabricated condition (*p* = 0.0005), 77% higher (63 ± 9.7% Z-oriented, 35.5 ± 4.1% X-oriented) in the HIP condition (*p* < 0.0001), and 56% higher (62.5 ± 14.8% Z-oriented, 40 ± 5.2% X-oriented) after vacuum-annealing (*p* = 0.0018). The elongation of the as-fabricated Z-oriented specimens approaches the expected values for annealed copper [11,39]. The mechanical properties of the as-fabricated specimens in Figure 6 are similar to those reported by Guschlbauer et al. [11,12] for specimens at 99.5% density and 188 ppm O_2_. Their reported as-fabricated samples had UTS of 231.6 MPa (X-oriented) and 177 MPa (Z-oriented), yield of 149.8 MPa (X-oriented) and 78 MPa (Z-oriented), and elongation ranging from 56.2–59.3%. The microhardness data in Figure 6D show a similar trend to the yield data. The mean data and confidence interval values do not exhibit strong separation between sample treatment or harvest orientations, where all mean values exceed 50 HV reported for annealed copper [39]. The hardness data are also similar to two reported hardness values of pure copper processed by EB-PBF. The hardness in this study is greater than that of Guschlbauer et al. reported at 57.3 to 57.8 (HV0.05) [12], whereas higher hardness reported by Ramirez et al. [36] at 88 (HV0.1) was possibly due to high oxygen content powder used in their study.

**Figure 7:**
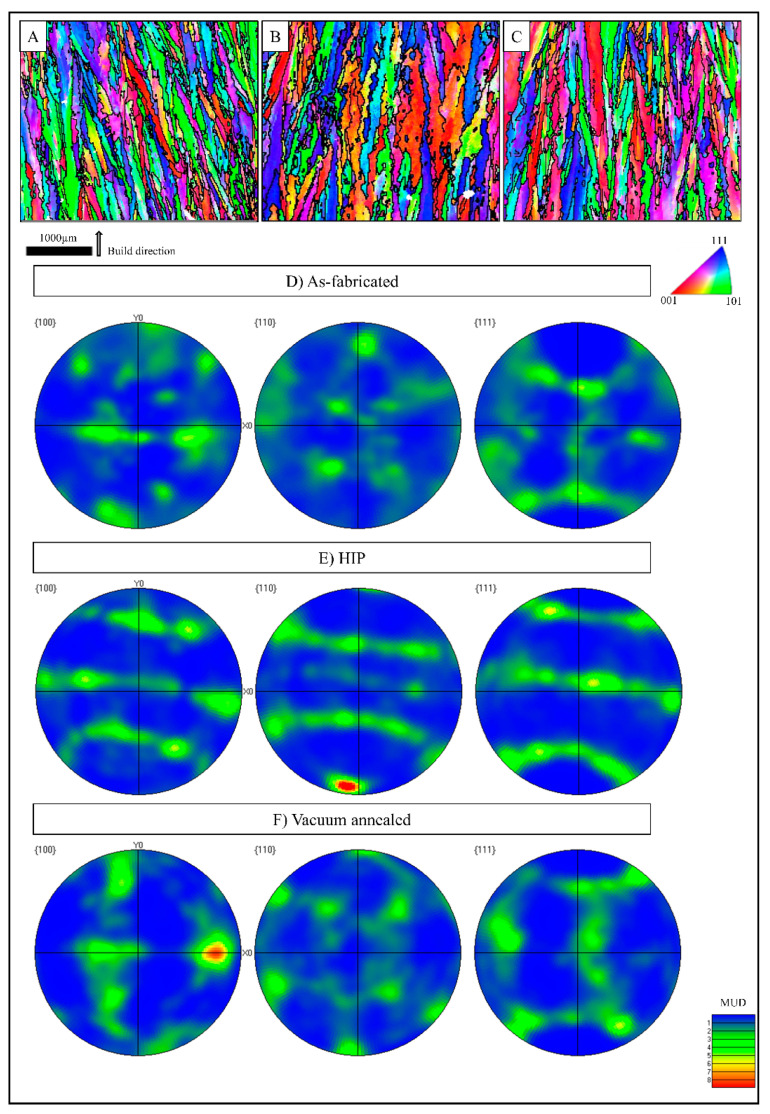
Inverse pole figure maps of as-fabricated, HIP, and vacuum-annealed specimens along the build direction shown with an arrow alongside the scale bar (**A**–**C**); pole figures of as-fabricated, HIP, and vacuum-annealed specimens (**D**–**F**), respectively. The color bar is multiple uniform density (MUD) for the pole figures ranging from 1–9.

For metals fabricated by processes which tend to form columnar grains or anisotropic microstructures such as by drawing, rolling, forging, or additive manufacturing, it is generally accepted that the properties differ by orientation due to the anisotropy [12,40,41,42,43,44,45,46,47]. Therefore, EBSD was performed on selected samples fabricated in this study to explore the influence of crystallographic texture of EB-PBF-processed unalloyed copper. Figure 7 shows reconstructed IPF maps and pole figures, respectively, for (A, D) as-fabricated, (B, E) HIP and (C, F) vacuum-annealed samples harvested in the X direction.

The reconstructed EBSD IPF maps in Figure 7A–C illustrate columnar grains and a mix of crystallographic orientations. The pole figures for (100), (110) and (111) planes in the as-fabricated X-oriented specimen show no strong texture at (100) projection in Figure 7D, which is often reported in AM-fabricated specimens, regardless of the material system or fabrication process [44]. The (110) and (111) pole figures also show no strong texture in the as-fabricated specimens. Guschlbauer et al. showed similar results where an IPF and orientation map with orientation distribution function figures for (100), (110) and (111) showed no observable crystallographic texture [11]. Figure 7E shows pole figures for the HIP condition, revealing an amount of imperfect texture in the <111> and <110> directions for the (100) projection and [$\bar{1}10$] texture in the (110) projection, however no heavily textured patterns were observed. The pole figures in Figure 7F for the vacuum-annealed condition reveal virtually no observable texture except for the <010> direction detected in the (100) projection. The yellow and red (6–8) MUD spots in Figure 7E,F are most likely large grains or localized clusters of sporadically measured planes and considered not representative of the entire fabricated specimens.

Most of the literature reported on mechanical properties of PBF and directed energy deposition (DED) are in a narrow material space, often focusing on commercially available alloys such as Ti-6Al-4V, IN718, 304 SS, 316L SS, Co-28Cr-6Mo, among others [44,45]. In pure metals and alloys produced by AM, many other factors influence strength and ductility in specimens exhibiting columnar grains and crystallographic anisotropy, which complicate the interpretation of property results. Studies across a number of alloy systems and AM processes report no clear trends in mechanical properties with test orientation and post-treatments where factors such as porosity, layer-induced defects, residual stress, oxidation, precipitation or allotropic phase changes, and localized cracking, among others, can have a significant impact on test results [40,44,45,46,48,49,50].

Since our study used unalloyed copper and samples were fabricated to high densities at temperatures exceeding 300 °C, some of the effects often reported in PBF and DED studies such as frequent layer defects, second phase precipitation, and micro-cracking become less influential. While no strong crystallographic texture was measured by EBSD in any of the X-orientation samples, columnar grains were clearly observed and may explain the differences in our mechanical properties, especially elongation and UTS. In a strain- and defect-free pure metal system, dislocation motion in the direction of long, thin grains would have more unobstructed movement in comparison with deformation across narrow grains, experiencing frequent grain boundaries. Therefore, the tensile samples harvested from the blocks in the build direction had significantly higher elongation as compared with the specimens harvested perpendicular to the build direction where fewer grain boundaries and oxides were present to impede deformation. The samples harvested in the X direction had consistently higher UTS compared with those in the Z direction due to significantly more interactions with grain boundaries and oxides during deformation, regardless of treatment. The yield was observed to be influenced by treatment conditions regardless of orientation and most likely is the result of any remaining residual stress which may resist initial deformation. These residual stresses, caused by the numerous thermo-mechanical cycles during the fabrication process, affect the bulk yield strength on a macro-scale where high yield strength values are reported for AM-processed materials with high internal residual stresses [45]. Heating close to 0.9 × *T_m_* was used in this study for HIP and vacuum-annealed samples and led to residual stress relief, thus resulting in lower yield strength and microhardness values, as shown in Figure 6B,D.

**Figure 8:**
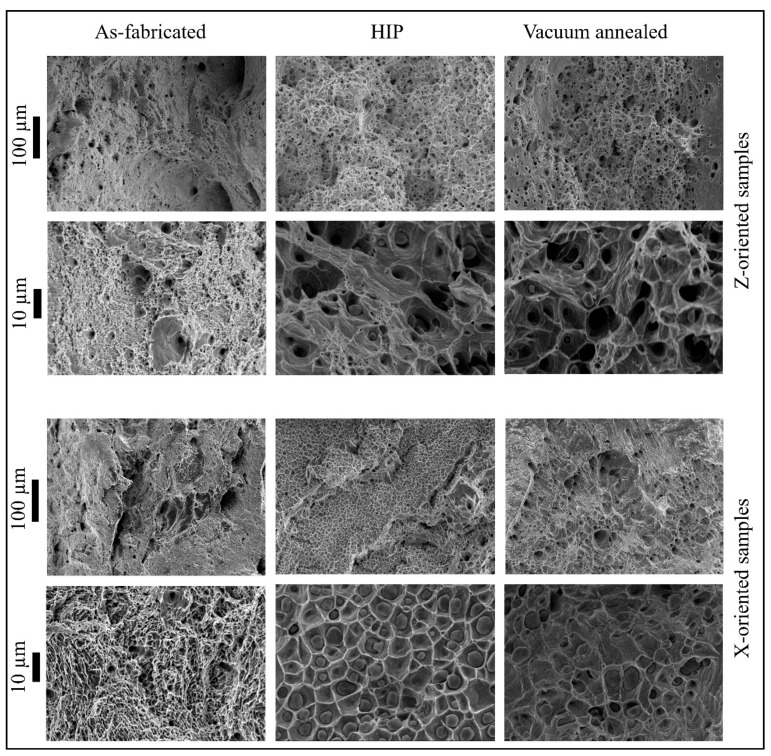
Fractographs of tensile specimens; top two rows: Z-oriented specimens (as-fabricated, HIP, and vacuum annealed); bottom two rows: X-oriented specimens (as-fabricated, HIP, and vacuum annealed); each scale bar is associated with the respective row.

Fracture surfaces were observed with SEM to explore the ductile fracture. Figure 8 shows representative examples of fracture surfaces for tensile specimens from each post-process condition for both harvesting orientations. The fractography indicates a transgranular fracture mode, forming the ductile dimples distinctive of soft materials like copper. In the case of the as-fabricated specimens, a shear zone at the outer edge is seen, followed by a ductile area in the middle of the sample. The ductile dimples are the manifestation of micro-void nucleation and their coalescence event, while the large size voids are the result of nucleated cavities or process-induced porosities [51]. Further, the number and distribution of shallow dimples are varied with respect to the orientation of the specimens, as observed especially in vacuum-annealed and HIP specimens. X-oriented samples show finer dimples more closely spaced than the Z-oriented specimens where plastic strain is higher, most likely due to the increased length of dislocation mean free path. However, the fractographs of the HIP specimens are markedly different from the as-fabricated conditions due to the presence of micron-scale oxides within many ductile dimples. This is notable because there was no difference in the measured oxygen content between the as-fabricated and HIP specimens, and it suggests that the high temperatures during HIP likely facilitated the mobility of oxygen and a subsequent growth of grain-boundary Cu_2_O particles. These large-scale oxides appear on the dimples due to the stress concentration and dislocation movement around them during the deformation process, similar to what was previously illustrated in as-fabricated EB-PBF copper by Guschlbauer et al. [11] and sintered cold-sprayed AM copper by Hutasoit et al. [52]. However, no feature of brittle failure such as cleavage facets was found in any type of specimen, suggesting that the incoherent copper oxides do not embrittle the copper parts at the concentration levels measured in the specimens. The vacuum-annealed samples also exhibited large-scale oxides compared to the as-fabricated specimens. However, these tended to be fewer and more widely dispersed along the grain boundaries than the oxides observed in the HIP samples. This is primarily attributed to the dissociation of oxide and low oxygen concentration measured in these specimens after vacuum annealing.

**Figure 9:**
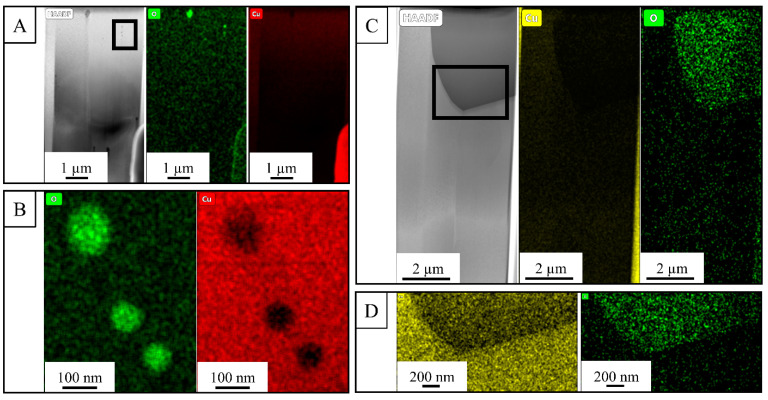
STEM EDS maps of an as-fabricated (**A**,**B**) and a HIP (**C**,**D**) specimen illustrating the size differences of copper oxide inclusions at the grain boundaries. Insets in (**A**) and (**C**) are shown in (**B**) and (**D**), respectively. Note the different length scales.

The STEM EDS maps in Figure 9A,B illustrate that the oxides are mostly at nano scales (50–200 nm) formed at the grain boundaries in the as-fabricated sample, whereas in Figure 9C,D, the oxide particles from the HIP specimen exhibit much larger sized scales (2–4 µm). This asserts the coalescence of nano-scale oxides as a result of heat treatment which, in turn, increases the interparticle spacing between these second phase oxides. This increased spacing between oxides in HIP and vacuum-annealed samples promotes the void sheet formation with increased void spacing, and favors higher ductility compared to the as-fabricated condition which has oxides at smaller nano-scale sizes with lower interparticle spacing [53].

## 4. Conclusions

In this study, the effects of different post-processing conditions on the mechanical properties of EB-PBF fabricated unalloyed copper are characterized and discussed. Rectangular blocks were fabricated with enabled automatic control settings from ~99.95% pure copper powder with a size distribution of 22–63 µm. Tensile specimens were extracted from these rectangular blocks with two different orientations and treated with HIP and vacuum-annealing alongside the as-fabricated condition. The following conclusions summarize the major findings of this study:

1. Mechanical properties of the as-fabricated specimens are observed to be similar to unalloyed annealed copper. X-oriented specimens have higher tensile strength values across post-processes than the Z-oriented samples with an average UTS of 211.2, 195.7 and 195.5 MPa in as-fabricated, HIP, and vacuum-annealing conditions, respectively. Maximum elongation to failure is recorded for Z-oriented vacuum-annealed and HIP specimens that showed average values of ~63%, as opposed to ~54% in as-fabricated samples.
2. With the density values reaching close to the theoretical density, the observed differences in UTS and elongation are proposed to be controlled by orientation anisotropy and interdependencies between the grain morphology and dislocation movement. Difference in yield strength is more pronounced between treatment conditions than the specimen orientation (e.g., 101.4, 87.3 and 56.7 MPa for X-oriented samples in as-fabricated, HIP, and vacuum-annealing condition, respectively) and is assumed to be affected by the differences in residual stress as a function of treatment conditions.
3. Density of the specimens increased slightly after the HIP treatment with an associated increase in ductility and decrease in strength. As expected, the vacuum-annealing process reduced the oxygen content within the specimen, also resulting in the lowest strength among all treatment conditions.
4. The EBSD maps show no apparent texture in the as-fabricated specimens, and a slight amount of texture in HIP and vacuum-annealed specimens. However, the effect of such texture in the mechanical properties is outweighed by morphological anisotropy due to the epitaxial columnar microstructure formed during the fabrication process.
5. While all treatment conditions show a ductile failure mode irrespective of the sample harvesting direction, HIP and vacuum-annealed specimens are noticeably different due to the presence of large micron-scale oxide particles within the dimples. It is suggested that the high temperature used in these post-processes promoted the coalescence and growth of grain boundary oxides which are mostly in the nano-scale size in the as-fabricated condition. This bigger oxide size led to larger interparticle distances and resulted in increased void spacing that, in turn, increased ductility in the HIP and vacuum-annealed condition.

## Figures and Tables

**Table 1:** Summary of the mechanical properties of BJ, L-PBF, and EB-PBF-processed copper (Best values are taken in each case; horizontal = loading transverse to build direction; vertical = loading parallel to build direction).

Process	UTS (MPa)	Yield Strength (MPa)	Elongation to Failure (%)	Relative Density (%)	Oxygen Content (wt. ppm)	Treatment Condition	Ref.
L-PBF	149	-	-	88.1	-	As-fabricated	[9]
L-PBF	248 ± 8.5	187 ± 5.3	9.2 ± 2.12	99.1 ± 0.5	446	As-fabricated	[10]
L-PBF	215 ± 7.2	51 ± 8.2	30 ± 3.04	-	446	Vacuum annealed	[10]
L-PBF	211 ± 4	122 ± 1	43 ± 3	99.3 ± 0.2	54	As-fabricated	[14]
EB-PBF	-	76	-	-	-	As-fabricated	[2]
EB-PBF	231.6 ± 5.4	149.8	56.2	>99.5	188	As-fabricated (horizontal)	[11]
EB-PBF	177 ± 3.3	78.1 ± 0.9	59.3 ± 7.5	>99.5	188	As-fabricated (vertical)	[12]
BJ	176.4 ± 6.5	N/A	67.2 ± 2.2	97.3 ± 0.1	N/A	Sintered, HIP	[13]

**Table 2:** EB-PBF melt parameters used to fabricate the specimens used in this study (the parameters shown are specifically those that have been modified from the standard commercial parameter sets for Ti6Al4V, V3.2 SP2).

Melting Parameter	Value
Surface Temp (°C)	600
Power Analyze Max Current (mA)	25
Power Analyze Min Current (mA)	8
Beam Speed (mm/s)	1000
Beam Current (mA)	8
Max Current (mA)	8
Focus Offset (mA)	18
Speed Function	20
Line Offset (mm)	0.13
Change for Each Depth (mm)	0.02
Heating Enable	TRUE
Max Heat Time (s)	5
Melt Heating Use Process Power	TRUE
Heating Between Models	TRUE

**Table 3:** EB-PBF preheat parameters used to fabricate the specimens used in this study (the parameters shown are specifically those that have been modified from the standard commercial parameter sets for Ti6Al4V, V3.2 SP2).

Parameter	Value
Focus Value (mA)	70
Focus Value Heater (mA)	150
Box Enable	TRUE
Box Size (mm)	85
Offset to Part (mm)	0.1
Max Current for Box (mA)	22.5
Jump Safe Sweep Max Current (mA)	13.5
Jump Safe Sweep Min Current (mA)	0.1
Jump Safe Sweep Speed (mm/s)	14600
Jump Safe Sweep Total Repetitions of Sweep	40
Jump Safe Sweep Max Number of Sweeps	40
Heating Enable	TRUE
Max Heat Time (s)	5
Heating Between Models	TRUE

**Table 4:** ICP-MS analysis of feedstock unalloyed copper powder prior to the first build. Values are reported in wt. ppm.

Ag	Cd	N	P	Pb	S	Sb	Sn	Zn
10	<1	2	5	2	8	<1	<1	<1

## Data Availability

Data supporting the findings of this study will be available upon request to the corresponding author (T.H.).
